# The influence of LLLT applied on applied on calvarial defect in rats under effect of cigarette smoke

**DOI:** 10.1590/1678-7757-2018-0621

**Published:** 2019-06-13

**Authors:** Camilla Magnoni Moretto NUNES, Camila Lopes FERREIRA, Daniella Vicensotto BERNARDO, Gabriel Barbosa OBLACK, Mariéllen LONGO, Mauro Pedrine SANTAMARIA, Maria Aparecida Neves JARDINI

**Affiliations:** 1 Universidade Estadual Paulista (UNESP), Instituto de Ciência e Tecnologia de São José dos Campos, curso de Odontologia, disciplina de Periodontia, São José dos Campos, São Paulo, Brasil.

**Keywords:** Laser therapy, Smoke, Bone regeneration

## Abstract

**Objective:**

Considering the global public health problem of smoking, which can negatively influence bone tissue repair, the aim of this study is to analyze the influence of photobiomodulation therapy (PBM) on calvaria defects created surgically in specimens under the effect of cigarette smoke and analyzed with use of histomorphometric and immunohistochemistry techniques.

**Methodology:**

Calvaria defects 4.1 mm in diameter were surgically created in the calvaria of 90-day-old rats (n=60) that were randomly divided into 4 experimental groups containing 15 animals each: control group (C), smoking group (S), laser group (L), and smoke associated with laser group (S+L). The animals were subjected to surgery for calvaria defects and underwent PBM, being evaluated at 21, 45, and 60 days post-surgery. The specimens were then processed for histomorphometric and immunohistochemistry analyses. The area of bone neoformation (ABN), percentage of bone neoformation (PBNF), and the remaining distance between the edges of the defects (D) were analyzed histometrically. Quantitative analysis of the TRAP immunolabeled cells was also performed. The data were subjected to analysis of variance (ANOVA) in conjunction with Tukey’s test to verify the statistical differences between groups (p<0.05).

**Results:**

The smoking group showed less ABN compared to the other experimental groups in all periods, and it also showed more D at 21 days compared to the remaining groups and at 45 days compared to the laser group. The smoking group showed a lower PNBF compared to the laser group in all experimental periods and compared to smoking combined with LLLT group at 21 days.

**Conclusions:**

PBM acted on bone biomodulation, thus stimulating new bone formation and compensating for the negative factor of smoking, which can be used as a supportive therapy during bone repair processes.

## Introduction

Bone healing is a complex process that involves multiple cellular and molecular events, and therefore requires a longer time for cells to completely regenerate the affected area in this tissue.^1^ Furthermore, there are several factors that can negatively influence bone tissue repair. A vast amount of literature shows smoking as having a primarily negative effect on bone tissue repair.^2^ ^-^ ^6^

Smoking is a public health problem worldwide. The World Health Organization (WHO) estimated that 10 million deaths will have occurred by 2020 as a result of tobacco-related diseases, given that 70% of these cases are to be reported in developed countries.^7^ César-Neto, et al.^2^ (2003) have shown that cigarette smoke inhalation is harmful to bone metabolism, suggesting that cigarette smoke constituents (toxic heavy metals, polychlorinated biphenyls, dioxins and polycyclic aromatic hydrocarbons) and nicotine can have a negative impact on bone wound healing. Other major negative effects of smoking are high rates of failure in clinical dental procedures, such as periodontal surgery, implants, and different types of bone grafts.^2^ ^,^ ^8^ ^-^ ^10^

Bone healing can be stimulated by applying various molecules, such as bone morphogenetic proteins (BMPs), physical stimuli, (e.g. ultrasonography or electromagnetic field) and, more recently, photobiomodulation therapy (PBM).^11^ PBM is a locally applied therapeutic modality that produces biostimulatory effects, and it is used as an adjuvant in tissue healing and repair processes.^12^ ^-^ ^14^ Its mechanism of action on bone repair is not yet well understood,^15^ although it has been regarded as promoting angiogenesis^16^ and an increase in *in situ* blood flow, thus increasing the supply of circulating cells, nutrition, oxygen and inorganic salts to the bone defect. This process stimulates cellular growth, such as fibroblasts that are related to collagen production^17^ ^,^ ^18^, through an increase in osteoblasts proliferation, viability and differentiation^14^ ^,^ ^19^ ^,^ ^20^, and promoting mitochondrial respiration and ATP synthesis.^21^ All these events can promote bone healing and mineralization.^21^ ^,^ ^22^

Several pre-clinical and clinical studies have evaluated the effects of different PBM protocols. It has been established that PBM increases bone formation in calvaria defects models in rats.^23^ ^,^ ^24^ Theodoro, et al.^25^ (2016) demonstrated that low level laser therapy also decreases bone loss in periodontal disease. Such studies conducted in animal models provide important information about the complex interaction of smoking on the treatment options for wound healing. In addition to preclinical data, some randomized clinical trials showed that different PBM protocols accelerated both gingival tissue^26^ and bone wound healing.^27^

To the best of our knowledge, there is no study showing the possible adjunctive effects of PBM protocols on bone wound healing when smoking is impairing bone repair. Thus, this study tested the hypothesis that PBM would be able to repair a calvaria defect even in the presence of cigarette smoke, through immunohistochemic and histometrically analysis.

## Methodology

### Animals and experimental groups

This study was approved by the Animal Studies Review Board of the Institute of Science and Technology, UNESP – Univ. Estadual Paulista, São José dos Campos, Brazil (02/2015) and followed the ARRIVE guidelines for reporting *in vivo* animal experiments. Sixty adult female Wistar (*Rattus norvegicus*, *albinus*) rats (weighing 250 to 300 g and 3 months-old) were kept in cages at room temperature with a 12-hour daylight cycle, fed with Guabi Nutrilabor^®^ (Mogiana Alimentos; São Paulo, SP, Brazil) and water *ad libitum* supplied by a qualified staff, at the animal house of Institute of Science and Technology, UNESP – Univ. Estadual Paulista, São José dos Campos, throughout the experimental period*.* The animals were randomized and allocated into 4 groups, control group (C), smoke group (S), laser group (L) and smoke associated with laser group (S+L).

The sample size equal to 10 per group and standard-deviation of 0.07 units (estimated value by pilot study), it was found that by using power analysis using Tukey test (5%) to compare averages, it was possible to detect a difference of 0.06 unit with a power test up to 80%.

### Smoke exposure

Fifteen days after adaptation, S animals were subjected to the cigarette smoke inhalation protocol described by César-Neto, et al.^2^ (2003). The animals were kept in a 45x25x20 cm^3^ acrylic box with a compartment for lit cigarettes and another one for the animals. The animals were subjected to an adaptation period in which they were exposed to cigarette smoke inhalation for 5 minutes on the first day, 6 minutes on the second day, and 7 minutes on the third day. Afterwards, they were exposed at the same time to the smoke of 10 cigarettes for 8 minutes, three times a day for 5 days a week. The cigarette brand used (MINISTER King Size Unique - Souza Cruz; Rio de Janeiro, RJ, Brazil) contained different tobacco blends, sugar, cigarette paper, 10 mg tar, 0.8 mg nicotine, and 10 mg carbon monoxide, according to the product label information. Nonsmoking animals were also placed in acrylic boxes of the same size for the same period in order to simulate the same conditions.

### Calvarial defects creation and photobiomodulation therapy session

Sixty days after the beginning of cigarette smoke inhalation, a single calibrated operator performed the surgical procedure of calvaria defects. The animals were anesthetized by intramuscular injection of xylazine (13 mg/kg - Rompun^®^, Bayer; São Paulo, SP, Brazil) and ketamine (33 mg/kg - Dopalen^®^, Agribands do Brasil Ltda; Paulinia, SP, Brazil). The frontal region was trichotomized and disinfected, and a linear incision was performed in the center of the calvaria, thus exposing the parietal bone. The defect was made by a trephine drill which is 4.1 mm in outer diameter (Neodent^®^; Curitiba, PR, Brazil) with an electric motor (Driller, BLM 600 Plus^®^; Carapicuíba, SP, Brazil) that allows speed control at 960 rpm, and cooling with constant sterile saline flow. This procedure was carried out with extremely care to avoid damage to the dura mater or the superior sagittal sinus. After removing the bone block and bleeding control, a single application of PBM was performed transoperatively. The PBM was applied on the edge of the bone defect by using a GaAlAs diode laser (DMC^®^ Equipamentos Ltda., São Carlos, SP, Brazil) with the following physical parameters: wavelength of 660 nm, energy density of 8 J/cm^2^, cross-section tip of 0.028 cm^2^, 30 mW continuous power feed, applied in a clockwise direction for 16 seconds on each of the four points, totaling 64 seconds. After the laser procedure, the sutures were performed with silk thread (4-0 Silk, J&J Ethicon^®^; São Paulo, SP, Brazil). After surgery, the animals were fed on a regular diet with water *ad libitum,* a single dose of 1 mg/kg intramuscular antibiotic (Penicillin G benzathine, Fort Dodge^®^, Saúde Animal Ltda; Campinas, SP, Brazil) and a single dose of 5 mg/kg of ketoprofen (Laboratório Teuto Brasileiro S.A.; Anápolis, GO, Brazil) by the subcutaneous route for the control of postoperative pain.

### Euthanasia and processing of the sample

The animals were anesthetized, followed by cardiac perfusion with 4% formalin on the following periods: 21, 45 and 60 days. For each period, 5 animals were euthanized per experimental group. The original surgical defect area and the surrounding tissues were removed in bloc and immersed in a 10% paraformaldehyde buffered solution during 48 hours, which were then demineralized with a 10% ethylene diamine tetraacetic solution (Dinâmica^®^ Química Contemporânea Ltda.; Diadema, SP, Brazil) at a pH of 7.8. After decalcification, the fragments were included towards the section surface in a paraffin block, thus obtaining serial sections of 5 µm, totaling ten blades with 10 sections per block that were stained with hematoxylin and eosin (H&E). Serial sections of 4 µm thickness were also performed for immunoistochemistry analysis for the marker tartrate-resistant acid phosphatase (TRAP).

### Histomorphometric analysis

The histological sections were scanned on Pannoramic Desk (3DHISTECH) and the images were obtained with use of Case Viewer 1.4 -3DHISTECH, being analyzed quantitatively by means of the Image J 1.31 application software (U. S. National Institutes of Health; Bethesda, Maryland, USA).

A single examiner captured the images that received codes which were subsequently analyzed by another examiner CMMN, thus allowing blinded data to be obtained. The histomorphometric measurement of the area of bone formation was calculated (in mm^2^) at 25x magnification for all groups (n=10 per group), and data were collected for statistical analysis.

The measures of two analyses with same structure were subjected to the Kappa test, and the examiner was considered calibrated (k=0.89).

### Histomorphometric parameters

For analyzing histomorphometric parameters, the original total area (TA) of the defect size was initially measured, thus identifying the external and internal surfaces of the calvaria as well as the right and left reversal lines where the bone was trephined, thus obtaining the following measurements:

- Area of bone neoformation (ABN): the area of new bone formation measurements from the right and left edges of the defect.

- Percentage of bone neoformation (PBNF): the total area was measured in square millimeters and was considered to represent 100% of the area to be analyzed. The newly formed bone area was also measured in square millimeters and calculated as a percentage of the total area.

- Distance between neoformed bone edges (D): remaining space between the edges of the neoformed bone within the defect, made by a linear measure.

### Imunohistochemistry analysis

The sections were dewaxed in xylol and alcohol baths. Antigenic recovery was performed in 10 mM sodium citrate solution, pH 6.0 and incubation in a pascal pressure cooker (Dako, USA). Endogenous peroxidase blockade occurred in 3% hydrogen peroxide solution for 30 min, followed by washing in PBS buffer (Phosphate Buffered Saline, pH 7.4) associated with Triton detergent for 5 minutes. After dilution of the primary antibody in commercial antibody diluent (Spring Bioscience; Fremont, California, USA - cod ADS-125) at 1:100 for TRAP (sc – 30833, Santa Cruz Biotechnology, Inc.; Santa Cruz, California, USA) the sections received the solution and were incubated overnight for 18 h at 4°C. After this time the excesses of the primary antibody from the slides were removed and washed with PBS. For the amplification of the reaction the cuts received the HRP polymer (Nichirei Biosciences Inc.; Tokyo, Japan - cod. 414154F) which was incubated for 30 minutes in a humid chamber. The slides were again washed in PBS for the reaction developing step. The liquid DAB (Spring Bioscience; Fremont, California, USA - cod DAB-125) was diluted in DAB diluent at a 1:50 ratio. This solution was applied over the cuts and allowed to soak for 3 to 5 minutes, with the slides being subsequently counterstained with hematoxylin. The sections were deparaffinized in xylol and rehydrated in alcohol.

The sections (n=5 per group) were analyzed with a light microscope Axiophot 2 (Carl Zeiss; Jena, Germany), and the images were captured by an AxioCam MRc 5 (Carl Zeiss; Jena, Germany), which transfers the captured images to the application software AxioVision Release 4.7.2. (Carl Zeiss; Jena, Germany). Quantitative analysis of TRAP immunolabeled cells was performed by a calibrated and blinded examiner (CMMN).

### Statistical analysis

The data were previously subjected to Shapiro Wilk test and the results indicated that the residuals were normally distributed and, by plotting against predicted values, uniformity was verified and no ANOVA assumptions were violated. Analysis of variance (two-way ANOVA) was performed to evaluate the relationship between the subgroups subjected to the treatment and sacrifice time.

The data were analyzed statistically with use of the application software GraphPad Prism (version 7.00 for Windows, GraphPad Software; La Jolla, California, USA) and Tukey’s multiple comparison test (α=5%).

## Results

### Quantitative and qualitative histological analysis

**Figure 1:**
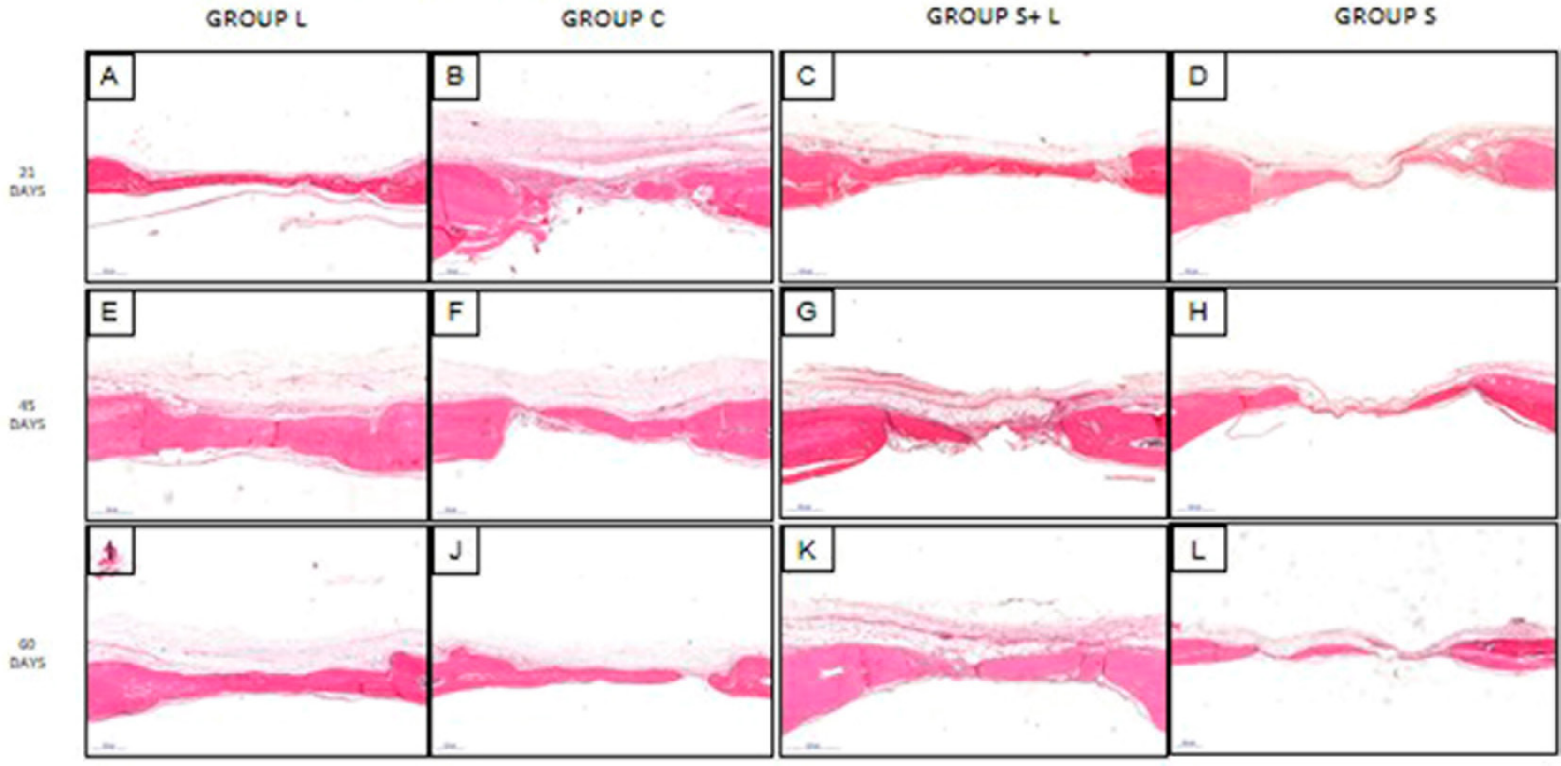
Photomicrography of histomorphometric analysis of groups on each experimental period. Group L (A, E and I); Group C (B, F and J); Group S+L (C, G and K); Group S (D, H and L) at 21, 45 and 60 days. Scale 500 μm and 2X magnification

The qualitative histological comparisons between groups are described below and resumed in the Figure 1, according to the analyzed periods.

### 21 days

In the L group, it is possible to observe the total covering of the wound by a narrow thickness of bone tissue with areas of immature bone tissue covered by a thin band of connective tissue that has been vascularized and cellularized, with thin collagen fibers parallel to the wound and throughout its length (Figure 1A). In group C, a bone formation can be observed in the wound edges which extend towards the center, but without closing it. A wide range of loose connective tissue richly vascularized and cellularized can be seen above the newly formed bone, and a thin layer of parallel collagen fibers that fill the area where there is no bone formation (Figure 1B). In the S+L group, there is a thin area of newly formed bone from the edges of the wound extending to the central portion, covering it almost entirely, showing characteristics of predominantly immature bone. The newly formed tissue is covered in its extension by a narrow band of loose connective tissue with thin collagen fibers, fibroblasts and blood vessels, which connects the bone tissue from the central region to the edge of the wound (Figure 1C). In the group S, small areas of bone neoformation were observed on the wound edges, interspersed by dense connective tissue, with thick collagen fibers parallel to each other interconnecting the newly formed bone tissues at the edges of the wound. In the region above the wound there is an extensive area of loose cellular and vascularized connective tissue and sparse inflammatory infiltrate (Figure 1D).

### 45 days

For the L group it is noted that the wound is completely covered by the bone tissue with characteristics of mature bone tissue. This tissue is thicker than in control and is covered at all times by a thick band of vascularized and cellularized loose connective tissue, with dense collagen fibers parallel to the neoformed bone tissue and throughout its length (Figure 1E). In group C, new bone formation is observed at the wound edges that extend along the defect, with small areas of bone neoformation, but without completely closing the defect. A wide range of richly vascularized and cellularized loose connective tissue is observed above the newly formed bone, and there is a thin layer of parallel collagen fibers that fill the area in which there is no bone formation (Figure 1F). In the S+L group, compared to the S group, there is a greater area of new bone formation from the wound edges that extend to the central portion, covering it almost completely, with characteristics of predominantly immature bone. The newly formed tissue was covered in its extension by a thick band of loose connective tissue with thin collagen fibers, fibroblasts and blood vessels, which connects the bone tissue of the central region to the edge of the wound (Figure 1G). In the S group were observed thin areas of bone formation with immature bone tissue in the wound edges and towards the center, but without closing the area. Note the presence of a narrow band of dense connective tissue, with collagen fibers parallel between the borders and connecting the areas of immature bone tissue, as well as a narrow area of richly vascularized and cellularized loose connective tissue above the dense collagen fibers (Figure 1H).

### 60 days

In group L, a complete closure of the wound by a tissue with a predominance of mature bone tissue characteristics and some regions of immature bone tissue characteristics. The thickness of neoformed bone tissue is greater than that of group C, and the loose connective tissue richly cellularized and vascularized with thin collagen fibers covers all its extension (Figure 1I). For group C, new bone formation is observed at the edges of the wound, at a lower thickness when compared to the group 45 days, which extends along the defect without complete closure of one border to the other. A long and narrow band of richly vascularized and cellularized loose connective tissue is observed above the newly formed bone, and a thin layer of parallel collagen fibers that fills the area where there is no bone formation (Figure 1J). For the S+L group, complete wound closure is observed by thick bone tissue, predominantly with mature bone features, covered by a thin layer of thick collagen fibers, fibroblasts and blood vessels, and by a thin layer of vascularized and cellularized loose connective tissue (Figure 1K). For the S group, thin areas of newly formed bone with immature bone tissue were observed on the edges and at the center of the wound, with thick collagen fibers and fibroblasts, blood vessels forming a cord connecting these bone tissues. Covering this entire region, an extensive but narrow band of richly cellularized and vascularized loose connective tissue with discrete inflammatory infiltrate could be observed (Figure 1L).

**Figure 2:**
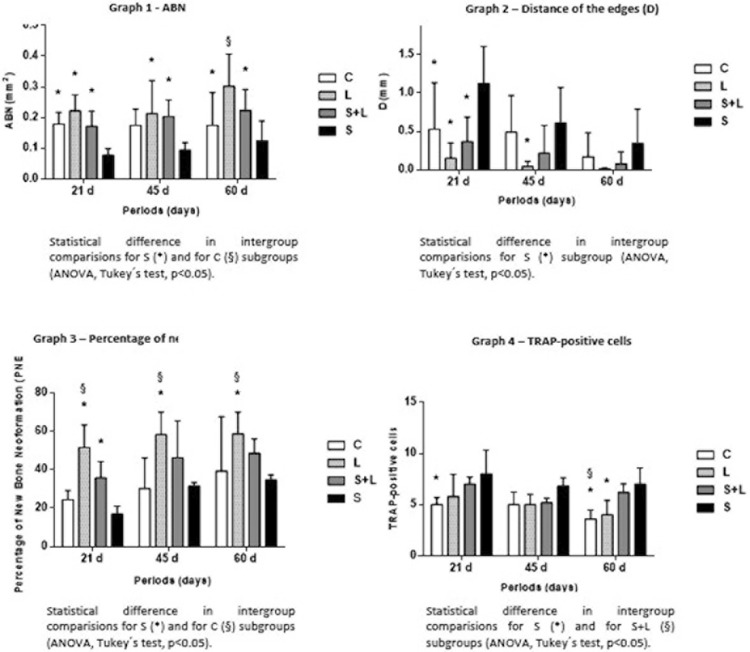
Graphics

The histomorphometric data analyzing the effect of the proposed LLLT session on bone repair for the ABN analysis (Figure 2, Graph 1) showed, in intergroup comparisons: at 21 days, for the comparison between the L and S groups(LxS; *p*=0.0002), for the comparison between the C and S groups (CxS; *p*=0.0135) and for the comparison between the S+L and S groups (S+LxS; *p*=0.0262); at 45 days, for the comparison between the L and S groups (LxS; *p*=0.0001) and for the comparison between the S+L and S groups (S+LxS; *p*=0.0067) and at 60 days, for the comparison between the C and S groups (CxS; *p*<0.0001) and for the comparison between the S+L and S groups (S+LxS; *p*=0.0189), wherein more newly formed bone tissues were observed at the edges of the wound compared to S, and was confirmed by the histological findings (Figure 1). The comparison between the L and C groups (LxC; *p*=0.0014) at 60 days showed a complete closure of the wound compared to C.

Graphic 1- Mean±SD of area of bone neoformation (ABN). Intergroup comparisons for all periods. *, significant difference compared to the S group at the same study time point (p<0.05). §, significant difference compared to the C group at the same study time point (p<0.05)

Graphic 2- Mean±SD of distance of the edges (D). Intergroup comparison for all periods. *, significant difference compared to the S group at the same study time point (p<0.05)

Graphic 3- Mean±SD of percentage of bone neoformation (PBNF). Intergroup comparison for all periods. *, significant difference compared to the S group at the same study time point (p<0.05); §, significant difference compared to the C group at the same study time point (p<0.05)

Graphic 4- Mean±SD of TRAP-positive cells. Intergroup comparison for all periods. *, significant difference compared to the S group at the same study time point (p<0.05); §, significant difference compared to the S+L group at the same study time point (p<0.05)

For the D parameter (Figure 2, Graph 2) a comparison between the C and S groups (CxS; *p*=0.0047), a comparison between the L and S groups (LxS; *p*<0.0001) and a comparison between the S+L and S groups (S+LxS; *p*=0.0002) showed a lower distance between the defect on the edges for groups compared to S at 21 days. The LxS (*p*=0.0078) comparison was also observed at 45 days.

The S group as compared to L showed small new bone formation percentages (Figure 2, Graph 3) for 21, 45 and 60 days (LxS; *p*<0.0001, 0.0004 and 0.0016, respectively). The comparison between S+LxS groups (*p*=0.0180) were also observed at 21 days, and the S group showed lower percentage of bone neoformation. The qualitative and quantitative data analysis showed the lowest bone percentage for the S group. For LxC, the same findings were noted at all evaluated periods (*p*=0.0003, *p*=0.0002 and *p*=0.0151, respectively) in which L revealed better results for this parameter compared to the C group.

According to statistical analysis, the S group had worse performance compared to the other groups at all evaluated parameters, whereas the L group showed better results when compared to the C group, confirmed by the qualitative histological findings.

### Immunohistochemical assessment

The S group showed a higher number of TRAP-positive cells when compared to group C on day 21 (*p*=0.0134). The animals of group S (*p*=0.0046) and group S+L (*p*=0.0369) showed a greater number of TRAP-positive cells when compared to the C group at 60 days. Group L showed a lower number of TRAP-positive cells when compared to group S at 60 days (*p*=0.0134) (Figure 2, Graph 4).

## Discussion

The objective of this study was to evaluate whether photobiomodulation therapy of the protocol proposed would reduce the known negative effects of smoking in bone repair of calvaria defects, and the established hypothesis was confirmed.

Experimental studies regarding the effects of low-level laser irradiation on human and animal cells in culture should represent an important pre-requisite to clinical trials in humans. The use of PBM in bone healing biostimulation has grown steadily, and studies showed positive results in bone healing. PBM leads to a cascade of photobiological events that may have advantages over healing, such as increased cell metabolism and collagen synthesis, especially with regard to the ability to modulate inflammation, accelerate cell proliferation, improve bone repair and promote the differentiation of mesenchymal cells and osteoblastic differentiation, also allowing a decrease in the number of osteoclasts.^23^ ^,^ ^24^

Furthermore, the negative effect of smoking on bone formation evidenced by a significant decrease in trabecular bone volume and trabecular thickness of the mineralized surface, rate of mineral apposition and new bone formation, reveal the negative effects of smoking^28^ and an increase in the number of osteoclasts. However, when combined with PBM, it was demonstrated to minimize the deleterious effects of smoking, especially on bone metabolism, hence regulating the RANK/RANKL/OPG system.^29^ ^,^ ^30^

In this study, smoking affected bone formation, resulting in a higher number of TRAP-positive cells in animals of the S group compared to the other groups, demonstrating that smoking inhibited cell proliferation and delayed the repair process that can be evidenced by the reabsorption activity with greater number of osteoclasts, as demonstrated by the immunolabeling for TRAP. PBM-treated animals showed a lower number of TRAP-positive cells relative to animals from the S group. These results reveal a potential effect of PBM on bone metabolism regulation under adverse conditions such as smoking. This fact justifies the improvement of the tissue repair process provided by PBM. In this way, recent studies have demonstrated similar results in rats under the action of smoking.^28^ ^,^ ^29^

In the analysis of new bone formation percentages, which revealed that a normal systemic situation associated with PBM (L subgroup), was higher from day 21 day until the 60 day, but other conditions, especially those with the condition of cigarette smoke inhalation, which initially had a lower formation percentage, had similar results on day 60. Thus, the smoke inhalation condition did not interfere with the repair process, and PBM was able to regulate the increase in osteoblastic activity^31^ by regulating the decrease in osteoclastic activity,^26^ demonstrating a favorable effect on bone structure.

When associated with PBM, the remaining distance between the edges of the defects under the effect of cigarette smoke inhalation presents a statistically significant difference only in the initial period of 21 days, which can be explained by the fact that PBM acts more effectively in the early stages of bone regeneration processes than in later stages due to increasing vascularization and early start of inflammatory response, which allows the healing proliferation stage to be reached sooner than it can be under smoke inhalation conditions.^32^ In addition, in our study, there was only one application of PBM similar to the studies by Cunha, et al.^32^ (2014), Almeida, et al.^33^ (2014) and Moreira, et al.^34^ (2018), who also observed greater initial bone formation compared to the control groups. In a protocol with higher number of PBM applications compared to our study, Oliveira, et al.^35^ (2008) obtained faster results in the repair process and attributed this fact to the number of sessions performed, since a longer period of treatment may intensify the process of bone differentiation and proliferation. Therefore, it is relevant to mention that not only choosing the PBM parameters but also considering the area and tissue of the defect to be treated are important in achieving the desired results, since the number of sessions may be related to the amount of stimulation delivered to the tissue, thus interfering in the final process result.

Chung, et al.^36^ (2012) concluded that the PBM can also indirectly influence in the activity of bone cells through the modulation of the transcription factor activity and the synthesis of the inducer protein. In response to the PBM, mitochondrial activity increases, and the reactive oxygen species that can upregulate genes related to cell behaviors, such as proliferation and migration, are released. Therefore, PBM can improve bone formation and bone resorption through a combination of mechanisms.

The PBM therapy effectively increases the area of newly formed bone at the edges of the defect in both the initial and late repair periods, as expected according to the literature.^15^ ^,^ ^24^ ^,^ ^36^ When associated with cigarette smoke inhalation, PBM presents better results than those of control, evidencing the effectiveness of PBM’s adjunct use in the repair of critical defects, even with the presence of smoking. Franco, et al.^37^ (2013) reported controversial results about the effect of passive smoking on the regeneration of rat femoral defects, they used stimulation with laser therapy and observed no significant difference in the volume of newly formed bone compared to the control group that was not subjected to laser therapy.

The use of laser on several tissues, including bone tissue in experimental models under other conditions,^13^ has been investigated and the literature has proven its stimulatory effects. However, the exact regulation mechanism by which the laser acts on the tissues is not fully understood,^38^ and therapy biomodulation depends on several factors, such as the wavelength being used, since tissue components can influence light scattering.^23^ In the present study, a single application of LLLT with l 660 nm, energy density of 8 J/cm^2^ was applied because recent studies of Garcia, et al.^23^ (2014) and Bosco, et al.^24^ (2016) demonstrated that LLLT with l 660nm laser presents biomodulatory effect on bone healing when used transoperatively at the borders of the surgical defect, corroborating the findings in our study. However, comparing the different doses, application protocols and experimental models between such studies is difficult.^24^ According to a review by Brassolatti, et al.^39^ (2018), the use of both red and infrared wavelengths in lasers investigated the promotion of osteogenic effects in defects considered critical, and suggests that the infrared wavelength in lasers is better due to their wavelength promoting a greater penetration depth in the tissue being treated. However, according to Tani, et. al.^40^ (2018), using red laser (635 nm) in osteoblasts induce increases in the formation of mineralized bone-like nodule structures and the expression of a critical regulator for different osteoblast processes including growth, migration, differentiation, as well as phenotype maintenance and upregulation of all tested osteogenic differentiation markers. However, in osteoblasts, the infrared laser (808 nm) upregulated neither the expression of OPN nor ALP.

These factors related to PBM parameters and the use of other methods such as micro CT for data analysis could help to explain the PBM benefits observed in this study.

Thus, this study can contribute with data on the PBM effects on bone tissue in the presence of smoking, which very often occurs in the case of adult patients who require procedures involving bone repair related to the Dentistry field.

## Conclusion

Within the limitations of this study, it can be concluded that the PBM protocol used provided adjunctive effect on osteogenesis and may compensate the negative factor of smoking in the bone repair process.
